# Using data calibration to reconcile outputs from different survey methods in long-term or large-scale studies

**DOI:** 10.1007/s10661-021-09727-2

**Published:** 2022-02-14

**Authors:** Christopher S. Jones, David H. Duncan, William K. Morris, Doug Robinson, Peter A. Vesk

**Affiliations:** 1 Department of Environment, Land, Water and Planning, Arthur Rylah Institute for Environmental Research, Heidelberg, VIC 3084 Australia; 2 School of BioSciences, The University of Melbourne, Parkville, VIC 3010 Australia; 3 Trust For Nature, Collins St, Melbourne, VIC 3000 Australia

**Keywords:** Data calibration, Model prediction, Monitoring, Vegetation change, Grazing, Double sampling

## Abstract

Understanding the impact of management interventions on the environment over decadal and longer timeframes is urgently required. Longitudinal or large-scale studies with consistent methods are best practice, but more commonly, small datasets with differing methods are used to achieve larger coverage. Changes in methods and interpretation affect our ability to understand data trends through time or across space, so an ability to understand and adjust for such discrepancies between datasets is important for applied ecologists. Calibration or double sampling is the key to unlocking the value from disparate datasets, allowing us to account for the differences between datasets while acknowledging the uncertainties. We use a case study of livestock grazing impacts on riparian vegetation in southeastern Australia to develop a flexible and powerful approach to this problem. Using double sampling, we estimated changes in vegetation attributes over a 12-year period using a pseudo-quantitative visual method as the starting point, and the same technique plus point-intercept survey for the second round. The disparate nature of the datasets produced uncertain estimates of change over time, but accounting for this uncertainty explicitly is precisely the objective and highlights the need to look more closely at this very common problem in environmental management, as well as the potential benefits of the double sampling approach.

## Introduction

Long-term ecological datasets are disproportionately valuable for understanding ecology and informing policy (Hughes et al., 2017). In the absence of long-term or large-scale datasets, comparisons between different datasets are often required for analysis through time and space. Ideally, the methods used to collect each dataset are the same, so that the data have the same errors and biases. However, it is common in in ecology (e.g., Gorrod & Keith, 2009; Okeke & Karnieli, 2006; Thompson, 2006) and other sciences (e.g., Rand & Logerwell, 2011; Straus & Gelles, 1986) that different methods are used, or different surveyors are used that have different biases and interpretations of a method. It is essential to acknowledge and account for any changes in methods that may have occurred for any comparisons of data (e.g., Magurran et al., 2010).

Researchers deal with this problem of dataset differences in various ways. In many cases, the data from one source are transformed. For example, when comparing continuous data to ordinal data, the midpoints of categories are commonly used as a “best guess” of the value on a continuous scale, such as for Braun-Blanquet vegetation cover categories (Pahl et al., 1997; Gabrey & Afton, 2000; Naugle et al., 2000; Gabrey et al., 2002; Poulin et al., 2002). Using category midpoints can produce highly inaccurate estimates when the categories are large, and neglects the uncertainty implicit in the ordinal score, and so a range of alternatives for use of these types of data have been proposed (e.g., Furman et al., 2018; McNellie et al., 2019).

For converting categorical or ordinal scores to continuous estimates, a set of approaches were described by Duncan and Vesk (2013). In those approaches, continuous values were treated as unknowns and estimated from a model using informative Bayesian prior distributions based on category ranges and/or distributions from an alternative data source. Increasing the data resolution improved the ability to estimate change from historical qualitative surveys, but without calibration, potential biases in each dataset are not accounted for.

Data calibration is another way to account for differences in datasets. A common approach to conduct calibration is using double sampling, where two datasets are collected at the same time using the methods being compared, and the two measures related. Typically, double sampling involves collecting a large sample-size, low-resolution dataset in conjunction with a higher resolution dataset on a subset of the same subjects (e.g., Collins, 2007). The larger dataset is calibrated by modelling the subset which was sampled twice and extrapolating to the rest of the low-resolution data (e.g., Collins, 2007; Eberhardt & Simmons, 1987; Harper et al., 2004). With calibration, one can simulate the dataset, with or without uncertainty, that might have been collected if higher resolution contemporary methods were used.

Accounting for the methodological differences between datasets can enable comparison of data through space and/or time. In this study, we sought to extend the approach of Duncan and Vesk (2013) to a calibration via double sampling. We used a case study of remnant native riparian vegetation along creeks in northern Victoria (Australia). The sites were originally surveyed in 1994–1995 (Robinson & Mann, 1996) using a visual, coarse categorical method, such as ordinal categories of tree counts of “none,” “some,” and “much.” The contemporary (2009–2010) surveys used a measured high-resolution method, such as counts on a continuous scale. Between the survey periods, some sites had a change in grazing management, while others were unchanged. Grazing has extensive and sometimes complex impacts on vegetation (Diaz et al., 2007; Milchunas & Lauenroth, 1993) and is a significant driver of riparian vegetation condition worldwide (Bryant et al., 1983; James et al., 1999). Determining the change in vegetation attributes in response to management conditions is required for validating previous investments and informing future management.

To facilitate the calibration, a subset of the sites surveyed in 2009–2010 was double sampled by employing both the contemporary and historical survey methods at the same time. Calibration was achieved by modeling the relationship between the two sets of double-sampled data. The calibrated version of the historical dataset was then compared with the high-resolution contemporary survey data to produce a superior estimate of vegetation change than would otherwise have been achievable. These results were contrasted with uncalibrated alternative approaches for estimating vegetation change using the informative Bayesian prior approach of Duncan and Vesk (2013).

By conducting double sampling of vegetation attributes, and modelling the differences between data from different survey methods, we aimed to (1) estimate a higher resolution version of the historical dataset using data calibration; (2) compare the modelled historical estimates to contemporary data to estimate vegetation change; (3) contrast these results to an alternative approach, using informative Bayesian priors; and (4) evaluate the approaches to inform their future use and the implications for our case study. We expected that the direction of vegetation change would be the same for both approaches but that the calibration approach would produce a less biased estimate of the magnitude of change.

## Materials and methods

### Study area

Field sites were located along the Broken, Boosey, and Nine Mile Creeks in northern Victoria, Australia. The creeks lie within a large floodplain and flooding events occur less than once every 10 years (CRCFE, 2001), but each of the creeks has highly modified flows due to installation of weirs and channelization of some banks. The surveys were conducted within creek frontages, which comprise the section of land between the baseflow edge of the creek and the adjacent property boundary. The spatial extent of each site was determined by boundaries delimited in a 1994/1995 study (Robinson & Mann, 1996). The frontages are mostly remnant native vegetation with many large trees remaining, although the vegetation has been highly modified by clearing, land use, and adjacent land use influences (Parks Victoria, 2006).

Most creek frontages within the study area are public land but many have been grazed by livestock under grazing licences since the late 1800s. As a result of that persistent grazing by livestock and other agricultural land uses, most of the creek frontages were categorized in the initial assessment as degraded, compared to expected benchmarks of habitat condition (Robinson & Mann, 1996). After this initial assessment, however, a land use review of the public land along these creeks led to the re-classification of much of the public land to “conservation reserve” in 2002, with the result that grazing by stock was removed from many creek frontages, and the boundaries of public land were fenced from adjacent private land, thus preventing other land use incursions into the public estate. These major changes in land-use and management between the initial surveys and contemporary surveys begged an analysis of the effects of these land use changes on the condition of the native vegetation.

### Survey methods

#### Historical survey

The historical study in 1994/1995 aimed to describe the native vegetation values along the creeks and suggesting management targets and actions for protection and restoration. The survey period ran from September 1994 until June 1995 with surveys commencing downstream and progressing upstream. Four hundred seventy-three survey sites were defined on both sides of the creeks based on site characteristics, vegetation type and/or condition, property boundaries, and adjacent land uses. The surveyors used visual estimation of vegetation and site variables, as opposed to measurement, to score vegetation condition for all 473 sites. The visual estimates were mostly scored on coarse categorical scales (Robinson & Mann, 1996) and were assessed for the whole site, with no subsamples (e.g., transects or plots) within sites.

#### Contemporary survey

Contemporary surveys were conducted between September 2009 and June 2010. The change from single visual assignment of a coarse categorical class per attribute (the technique applied at 437 sites) to high intensity point intercept sampling with replication, meant that 180 of the 473 pre-defined sites could be surveyed, providing a substantial dataset for the study. Overall, the sampling order followed a sequence of downstream sites first (in spring) to upstream sites last (winter) with no surveys done in January (austral summer). To avoid a direct correlation between distance upstream and season, just over one third of the sites were surveyed out of sequence.

Vegetation attributes were measured using repeated point quadrats (points) spaced at 50-cm intervals along line transects across each site, as used in other understorey vegetation studies, e.g., Bullock (1996), and are an effective method for sampling understorey vegetation cover (Floyd & Anderson, 1987; Goodall, 1952). Transects were laid out perpendicular to the creek edge; the length (median: 32.5 m, range: 6–173 m) and number of transects (mean: 5.2, range: 2–8) varied with the size and shape of the sites to account for the wide range of site areas (0.5–19.9 ha). The number of point quadrats per site varied substantially (median: 382, range: 105–846). Fewer points will result in decreased precision of the cover estimate for a given life form, which is exacerbated for life forms with low cover (Goodall, 1952). At each point, a 0.5-cm-diameter vertical rod was placed and both the ground surface type (bare ground, road, rock, log, or litter) and any plant part attached to a living plant that touched the rod (up to 5 m tall) was scored as present. Plants were categorized by life form (herb, tufted/non-tufted graminoid, shrub, geophyte, or fern), and life history (annual/perennial), and origin (exotic/native).

Tree densities were surveyed in belt quadrats, located on each transect, 2 m either side (4 m wide), and as long as each transect. All trees were identified and their diameter at breast height (DBH) recorded at 1.3 m from the ground (if taller than 1.3 m). Tree recruits were those shorter than 1.3 m.

#### Calibration survey

To calibrate the historical dataset, a subset of sites included in the contemporary survey were resurveyed using the historical survey method. The number of sites was limited by availability of the surveyor, but a stratified selection of 60 of the 180 (one third) were able to be double sampled sites. The resurveys and contemporary surveys for each site were done within 2 weeks of each other to avoid effects of seasonal change on survey results. To reduce observer bias, the resurveys were done by one of the original surveyors of the historical study (D. Robinson). We assumed that for the historical method surveys, the observer’s approach did not change between survey periods. These surveys were conducted in November and December 2009 and May to June 2010.

### Data analysis to evaluate change over time

Two separate analyses of the same data were conducted using two hierarchical Bayesian model types: calibration and non-calibration. Calibration used the additional double sampled data to determine the relationship between the sampling methods and scores and then used this to convert historical data to a scale equivalent to contemporary values. The non-calibration approach did not use the double sampled data and instead only used Bayesian priors to refine the historical scores. Models for both approaches were fitted using Bayesian inference employing Markov chain Monte Carlo (MCMC) methods with the open-source software package JAGS version 3.3.0 (Plummer 2003) in the statistical software environment R version 2.15.2 (R Development Core Team, 2012) with the package R2jags (Su and Yajima 2012).

#### Target vegetation attributes

Three variables were examined: nativeness, medium tree density and tree recruitment density to develop the models and the analysis approach. Nativeness is a percentage scale that describes the composition of understorey vegetation cover, i.e., the proportion of all vegetation cover that is native as opposed to exotic. This understorey attribute was used because it indicates the level of degradation due to exotic species invasion. The nativeness attribute closely matches the historical scoring, specifically the “mostly native” category, so this was used as the source attribute from the historical data. Nativeness and tree recruitment density are attributes that were more likely to have changed between the survey periods due to the relatively rapid growth rates of understorey life forms and tree seedlings, and sensitivity to grazing, whereas medium tree density was likely to change much less. The variables and the survey method are detailed in Table 1. Detailed model descriptions relevant to the study are provided in the Appendix.

**Table 1:** The scoring systems used in the historical and contemporary surveys for three selected vegetation attributes. Historical method details taken from Robinson and Mann (1996)

Attribute	Historical scoring system	Contemporary scoring system
Ground cover: Nativeness (the percentage of vegetation cover that is native, as opposed to exotic/weedy)	Four parts sum to 100%, each part estimated to the nearest 5% 1. Mostly native % (the percent area with mostly native vegetation) 2. Mostly weedy % (the percent area with mostly weedy vegetation) 3. 50/50% (the percent area with equal parts native and weedy vegetation) 4. Bare ground %	Point quadrats along a series of transects for each site. Percentage cover measured as the number of “hits” at each point for a given life form divided by the total number of points at each site, times 100 Nativeness is measured as percentage of plant “hits” that are native as opposed to exotic
Medium tree density (trees 25–50 cm DBH)	0 = None 1 = Some (< 40 per ha) 2 = Much (> 40 per ha)	Counts of the numbers of trees within a series of quadrats at each site, with a known area to enable density estimation per hectare
Tree recruitment density (trees < 2 m tall)	0 = None 1 = Some (< 50 per ha) 2 = Much (> 50 per ha)	Counts of the numbers of trees within a series of quadrats at each site, with a known area to enable density estimation per hectare

For the subjective historical data on tree recruitment density, recruits were defined as those < 2 m tall. Because tree height was not surveyed in the contemporary study, recruits were defined as trees with < 2 cm DBH, which corresponded roughly with trees < 2 m tall in the study area (Jones, personal observation).

#### Bayesian informative priors

The analysis included a set of Bayesian priors described by Duncan and Vesk (2013), which treated the historical quantitative variables as unknowns to be estimated with uncertainty (see Gelman & Hill, 2007 or McCarthy, 2007). In this study, we have changed the names of the Bayesian priors from “range,” “distribution,” and “combination” (Duncan & Vesk, 2013) to “uniform,” “normal,” and “truncated normal” to reflect their type more accurately. Duncan and Vesk (2013) compared three alternative forms of informative prior: uniform distributions over the ranges of values implied by the categories (“uniform”); normal distributions equivalent to the distributions of the objective contemporary survey data (“normal”); and a combination of the two approaches, i.e., a distribution truncated by the categorical range (“truncated normal”). In addition to these three forms of priors, a fourth option was added, which simply used the category midpoint of the original historical score. This “midpoint” approach is common in ecological studies that want to compare between categorical data across sites in space or time (Gabrey & Afton, 2000; Gabrey et al., 2002; Naugle et al., 2000; Pahl et al., 1997; Poulin et al., 2002). The midpoint is a prior that admits no uncertainty about the estimate. The use of these four prior forms was evaluated in both the calibration and non-calibration models. Standard uninformative (flat) priors (Gelman & Hill, 2007) were also used within the models where noted.

The uniform prior implies the unlikely assumption that an observer always correctly classifies attributes among categories. One option to account for misclassification errors is to have overlapping ranges. Overlapping ranges were tested but they made little difference to the estimates. For simplicity, we excluded these analyses here.

Normal priors were not expected to perform particularly well in this study since the distribution was derived from the contemporary data, which meant that the historical predictions were almost identical to the data. This prior would influence the resultant variable to a greater extent if the distribution was formed from an external data source, as was the case in Duncan and Vesk (2013). Models containing normal priors were presented for non-calibration results but were excluded for calibration results due to the difficulty in model estimation of the Pearson’s regression coefficients and the unknown state variable for each site, without any additional prior information.

#### Non-calibration (Bayesian reinterpretation) approach

The non-calibration model structure is like that of the calibration approach, but with the calibration sub-model excluded. Unlike the calibration approach, the response variable for nativeness was a normally distributed cloglog transformed proportion of cover. Proportions of cover were derived from the number of vegetation hits from the number of points sampled at each site. The response data for medium tree density and tree recruitment density were again modeled as a Poisson distributed variable.

A cloglog link function was used for the proportions, with normally distributed errors. Counts were Poisson distributed, and log transformed densities were modelled to estimate change. Because projective foliage cover of understorey life forms can vary seasonally, the change in cover was modelled as a function of true change and the difference (in days) in the time of year between the subjective and objective surveys.

#### Calibration approach to estimate change

**Fig. 1:**
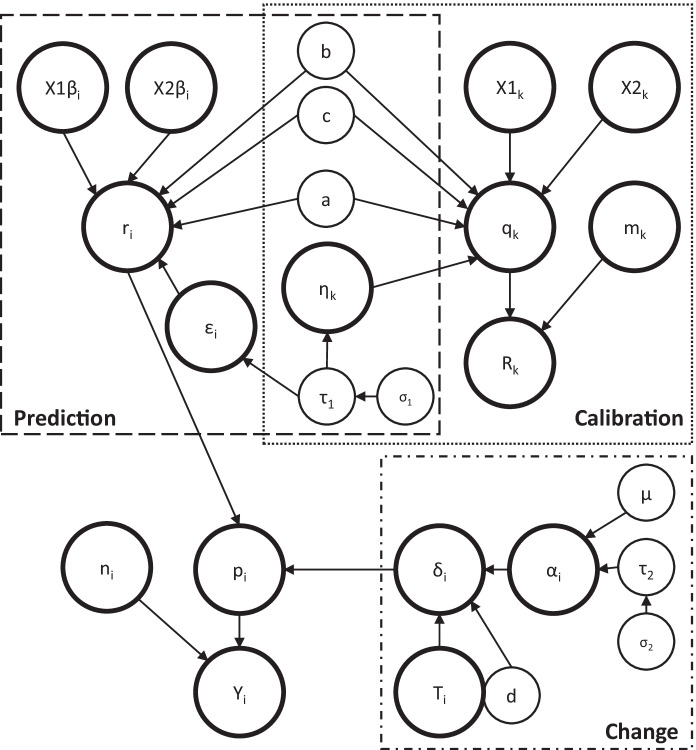
A directed acyclic graph, showing the structure of the binomial calibration-change model. Circles represent nodes of the model and arrows indicate casual dependencies between nodes. The three sub models are indicated in rectangles, the Calibration sub-model (dotted), the Prediction sub model (dashed) and the Change sub model (dot-dashed). *Y*_*i*_ is the observed number of sample hits at the *i*th site from a possible number of points, *n*_*i*_, with probability, *p*_*i*_. Calibration data are a subset, *k* of the dataset, *i*. The calibration of the historical data to contemporary method hits, *R*_*k*_, from the *k*th site is binomially distributed with probability, *q*_*k*_, and sample size, *m*_*k*_. The cloglog transformation of, *q*_*k*_, is a linear function of contemporary subjective vegetation predictor variables, (X1_k_, X2_k_) with coefficients (b, c), between-site variation, *η*_*k*_, and *ε*_*i*_, and intercept, *a*. The prediction sub-model uses the same function, intercept and coefficients but includes the historical subjective predictor data (X1β_i_, X2β_i_) to predict the historical probability of a hit *r*_*i*_. The change in vegetation condition, *δ*_*i*_, for any given site is a combination of the raw change, *α*_*i*_, and the difference in season between surveys, T_i_, where α_i_ is normally distributed with the mean change, *μ*, and the standard deviation of change, σ_2_

Expanding upon the non-calibration approach, we reinterpreted subjective data using Bayesian informative priors within a calibration-by-double sampling approach. The model is summarized with a directed acyclic graph (DAG, see Lauritzen & Spiegelhalter, 1988) in Fig. 1. The approach contains three steps, or sub-models: calibration, prediction, and change:

1. Calibration: Build models relating a contemporary, objective response variable to a selection of calibration survey (Table 1) and site predictor variables (Table 2).
2. Prediction: Generate calibrated data for the historical dataset.
3. Change: Infer change over time by comparing calibrated historical data and contemporary data.

**Table 2:** Details of site predictor variables for both the historical and contemporary surveys

Variable	Survey method	Details
Distance upstream	GIS mapping	Distance in km along the creek from the Murray River
Season (“days”)	-	Difference in days of the year between the survey date of the same site in the subjective and objective surveys
Creek	GIS mapping	The identity of the creek adjacent to that section
Area	Scaled parish plans	The area in hectares of each section
Road	Site observation	A binary score of the presence of a road within each section
Cropping/grazing	Site observation	A binary score for each adjacent land use
Water	Site observation	A binary score of the presence or not of water in the creek adjacent to each section

#### Calibration sub-model

The calibration sub-model calibrates the historical data by modelling the relationship between the data collected using both methods at the 60 double sampling sites. The response variables derived from the survey data have different forms depending on the sampling method used to measure them (Table 1). The form of each model depends on the type of response data. For example, the contemporary response variable “nativeness” was derived from point quadrat sampling along transects, is binomially distributed as number of “hits,” or success (where a life form is present at a point) from the total number of “points,” or trials. The data are entered into the model in the form of hits from points rather than simplifying to a proportion to account for the variable number of points surveyed in each section.

The change in nativeness can then be derived from modelling the change in the probability of hits over time. The response data for medium tree density and tree recruitment density were modeled as Poisson distributed count data using a log link function.

A complementary log–log (cloglog) link function was used to transform the nativeness probabilities (McCulloch & Nelder, 1989), allowing the cloglog probability to be modelled as a linear function of the calibration data predictors. The cloglog link was used in preference to logit because the logit link yielded poor convergence of MCMC chains.

Predictor variables were the subjectively surveyed vegetation attributes (Table 1), and a range of either environmental or site predictor variables (Table 2). The environmental data were centred and scaled by dividing by two standard deviations, to help models converge (Gelman & Hill, 2007), but were otherwise unmodified. Selection of predictor variables used in each calibration sub-model employed a multi-stage process:

1. Identify influential predictors by fitting Boosted Regression Tree (BRT) models to the data (Elith et al., 2008).
2. Fit generalized linear models (GLMs) with the most influential candidate predictor variables, and used Akaike information criterion (AIC) to evaluate the model fit.
3. Finally, use the candidate predictors in the models fitted with MCMC and compared the deviance, deviance information criterion (DIC) to select the final set of variables used (Pettitt et al., 2006; Spiegelhalter et al., 2002).

#### Prediction sub-model

The prediction sub-model used the same function, intercept and coefficients from the calibration sub-model, but using historical (Table 1) and site (Table 2) predictor data to predict the cloglog transformed probability of a hit (Fig. 1). Historical predictors were log-transformed densities, and cloglog transformed cover values, as per the calibration sub-model. Site data were again centred and scaled by dividing by two standard deviations (Gelman & Hill, 2007). The use of Bayesian priors in the prediction sub-model was the same as those used in the calibration sub-model.

#### Change sub-model

For a given response variable, the contemporary probability of a hit is equal to the calibrated past probability, plus some degree of change. Because seasonal variation influences many vegetation attributes, the difference in time of year between surveys was included in the model for change over time with a coefficient.

#### Comparisons of calibration and non-calibration models

The predictive performance of the calibration and non-calibration models were compared using cross-validation (Arlot, 2010) for each of the informative priors. A randomly selected ten percent of sites were held out for each fold. Predicted and observed values were compared to assess model performance. Performance of the calibration model was analyzed in two parts: first, by assessing the alternative forms of the calibration sub-model, and second, assessing each of the priors’ effect on the predictions. Pearson’s correlation coefficients of predictions and observations were calculated and the 95% confidence intervals of these correlations were compared for each model using statistical software environment R version 2.15.2 (R Development Core Team, 2012) with the function “*cor.test*” to assess model performance.

#### Evaluating grazing management influence on vegetation change

Following development of the different models on the three target attributes, the models were used to evaluate the impacts of grazing management. Livestock grazing is managed within the study area using grazing licences and fencing. To examine the effect of a change in management status, i.e., fenced or licenced, the estimated vegetation change between sites with alternative management states were compared (i.e., fenced and licenced). Two scenarios were contrasted, where management states remained the same between the two periods (unchanged), with three that changed (Table 3). Sites that were not licenced and were fenced at both the historical and contemporary survey periods, are the combination likely to experience the least grazing pressure and therefore expected to have the best vegetation condition. In contrast, those sites that were licenced and not fenced during both surveys had the greatest potential for grazing throughout and were expected to have the worst vegetation condition. To examine the effect of a change in management, sites where a fence was installed or a licence was revoked, or both were compared. Sites that had a licence revoked were expected to have the largest change in understorey vegetation, while sites that had no change in management or were fenced but remained licenced were expected to have the least change in understorey vegetation.

**Table 3:** Management combinations assessed in the study and their expected ranking of vegetation condition and magnitude of vegetation change. Each management combination is listed as having a change in management or no change

Management	Change or unchanged	Expected condition ranking	Expected magnitude of change
No licence, fence	Unchanged	1	Low
Revoke licence, fence	Changed	2	Moderate
Revoke licence, install fence	Changed	3	High
Licence, install fence	Changed	4	Low
Licence, no fence	Unchanged	5	Low

## Results

The results follow a sequence from model testing to model outputs and finally comparisons between the two model forms. The calibration model is then used to evaluate a livestock grazing impact on vegetation attributes.

### Non-calibration approach outcome

**Fig. 2:**
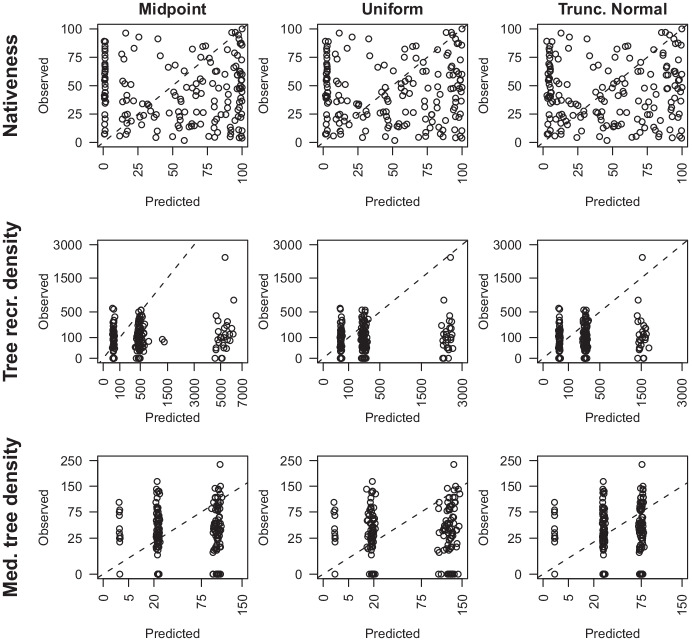
Cross-validation results of contemporary, objective values for each response variable across from non-calibration models with prior distributions, midpoint, uniform, and truncated normal. Tree density (stems per ha) figures are plotted on a square root scale. Dashed lines follow a 1:1 line (*n* = 170)

Cross-validation of the predictive performance of the non-calibration models for each response variable showed poor correlations between predicted and observed data (Fig. 2). Predictions for tree density closely conformed to the midpoints or implied categorical ranges of the subjective survey method. Nativeness predictions were less constrained due to the far greater number of categories however, there was no relationship between predicted and observed values. In contrast with the calibrated change model, these predictions spanned much greater ranges and tended to over-predict low values and under-predict high values (except for nativeness).

**Fig. 3:**
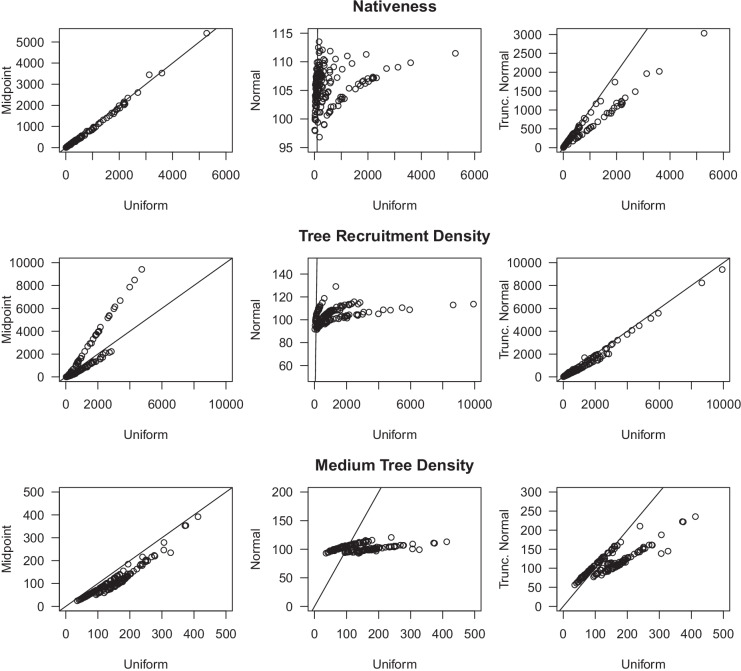
Comparisons of posterior values for the change, *α*_*we*_, as a percentage of the historical score, for each variable from alternative non-calibration models using prior distributions, midpoint, uniform, and truncated normal, for historical data. The dashed line is a 1:1 line

The percentage change estimates for each response variable were similar between non-calibration models with midpoint, uniform, and truncated normal priors (Fig. 3). Of these three prior forms, the truncated normal prior produced the most conservative range of values in the posterior. Results from normal priors were markedly different form other prior forms and produced a very narrow range of values. Because the true magnitude of change is unknown, evaluating these estimates is difficult. Such a narrow range of predictions for the Normal prior is unrealistic considering the potential for change in nativeness and tree recruitment density. We expected at least some sites to show large percentage changes for these attributes. The narrow range is precisely what was expected for the medium tree density. Distinctively different relationships were seen for sites based on the midpoint priors, as illustrated by divergent trends of points in Fig. 3. The largest, and unrealistic, predictions of percentage change were those from sites where the historical value was zero. Adding a small constant to these observed historical values improved predictions although the models predicted large values or different patterns for these values in some cases. A clear example of this is the midpoint and range comparison for tree recruitment density in Fig. 3.

**Fig. 4:**
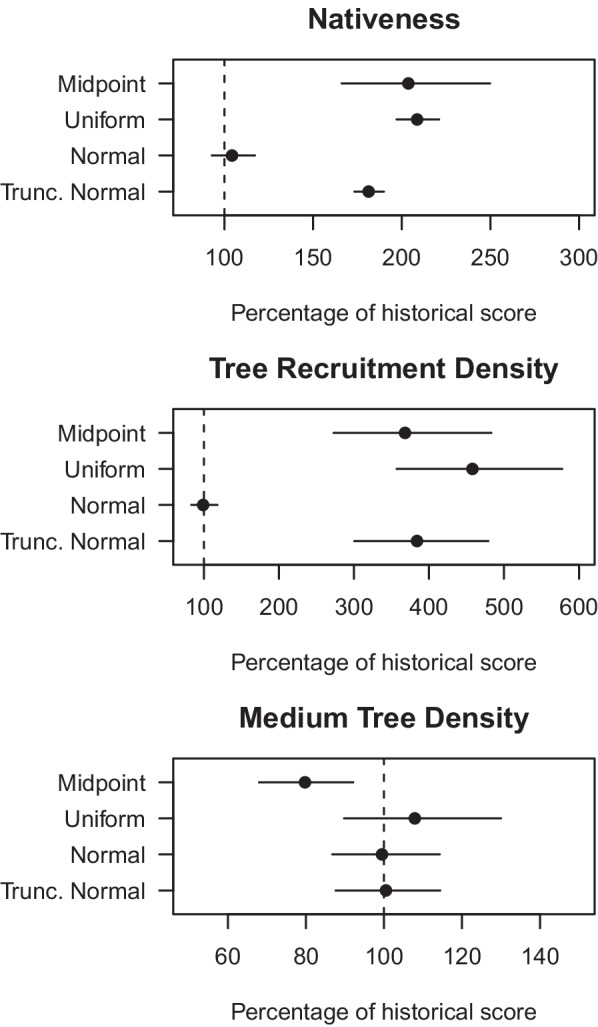
Comparisons of mean change, *μ*, across all sites from the non-calibrated approach for each vegetation attribute using alternative priors for the historical data from the model. Error bars are 95% credible intervals. The dashed line is located at the 100% percentage change level, i.e., indicating no change

Predictions of change from the normal priors showed no change, as was expected because the historical posterior estimates were modelled using the same mean and standard deviation of the contemporary sites. If the prior distributions were generated from an independent dataset as per Duncan and Vesk (2013) then use of this prior may have determined a change. The models with Normal priors were so constrained by the prior that it was uninformative about change and therefore excluded from the subsequent analyses. Across the different priors, sites on average had a ~ 200% increase in nativeness, ~ 400% increase in tree recruitment density, and no change in the medium tree density (Fig. 4). These large estimates of change were higher than expected due to the occurrence of very large estimates at some sites.

### Building a calibration model

**Fig. 5:**
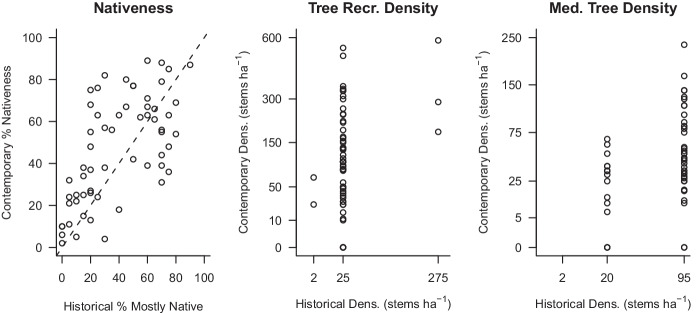
Relationship between the subjective, historical method score and the objective, contemporary method score, surveyed at the same time, i.e., double sampling, for the calibration surveys (*n* = 60). The dashed line is a 1:1 line. Historical densities are shown on a square root scale for visual clarity

The double sampled attributes used in the calibration sub-model were positively correlated with their counterparts (Fig. 5). Pearson’s correlation coefficients for nativeness, tree recruitment density and medium tree density were: 0.63, 0.36, and 0.32, respectively. The optimal set of predictor variables was as follows: “mostly native” and “mostly weedy” for the nativeness response model; “recruits” and “distance” for the recruitment density response model; and “medium trees” and “distance” for the medium tree density response model (Table 4). A site random effect, η, accounted for non-independence (Table 4).

**Table 4:** A summary of model selection for each calibration sub-model. Deviance information criterion was used to identify parsimonious models. The selected best models are shaded. *n* = 60

Response	Covariates included	Dhat	DIC	pD
Nativeness	Intercept (null)	2642.9	2818.9	176.0
	Mostly native + η	1258.4	1499.2	240.8
	Mostly native + mostly weedy + η	1258.9	1498.3	239.4
Recruit density	Intercept (null)	1562.0	1740.8	178.7
(< 2 cm DBH)	Recruits + η	1070.3	1312.7	242.4
	Recruits + distance + η	1070.4	1309.9	239.5
Tree density	Intercept (null)	963.2	1125.3	162.1
(25–50 cm DBH)	Medium trees + η	873.6	1087.2	213.6
	Medium trees + distance + η	872.6	1081.2	208.7

### Calibration approach outcome

#### Calibration sub-model

**Fig. 6:**
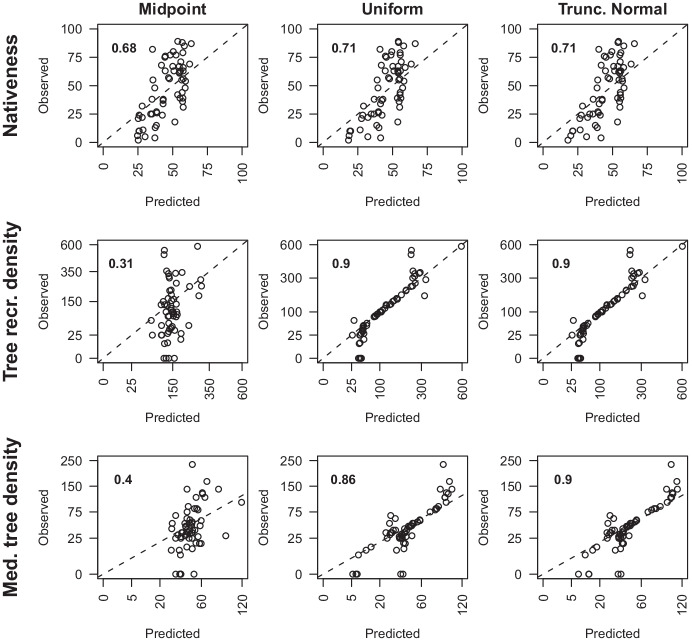
The predictions versus the observations from calibration sub-models for the held-out data from 10-fold cross-validation of nativeness (percentage), tree recruitment density (stems per ha), and medium tree density (stems per ha) (*n* = 60). Cross-validation of the calibration sub-model was run within the whole calibration change model. The three prior distributions, midpoint, uniform, and truncated normal, were assessed for each response variable. Tree density figures are plotted on a square root scale. Bold numbers in the top-left of each plot are the Pearson’s correlation coefficients. Dashed lines represent the 1:1 ratio

Cross validation of calibration sub-models for each vegetation attribute showed they generally performed relatively poorly for Midpoint priors when predicting to new sites due to the lack of information to inform the value of η (site discrepancy) at new sites (Fig. 6). Models for nativeness performed better than for the other response variables due to the larger number of categories for the variable, i.e., 5% intervals for historical understorey categories compared to three density classes for trees. But, the predictions were constrained near the mean due to generally conservative estimates of η or a lack of explanatory power in the covariates, and they were not substantially better for the Uniform or Truncated Normal priors, with Pearson’s correlation coefficients: 0.68, 0.71, and 0.71, respectively. The categorical variables of density were worse for the Midpoint prior but predicted relatively well using the Uniform and Truncated Normal priors (r > 0.85) (Fig. 6). The Uniform and Truncated Normal priors produced very similar predictions for all sites and all three response variables. Within each of these results there is a general pattern of over-prediction of low values and under-prediction of high values; this is particularly evident for nativeness.

#### Prediction sub-model

**Fig. 7:**
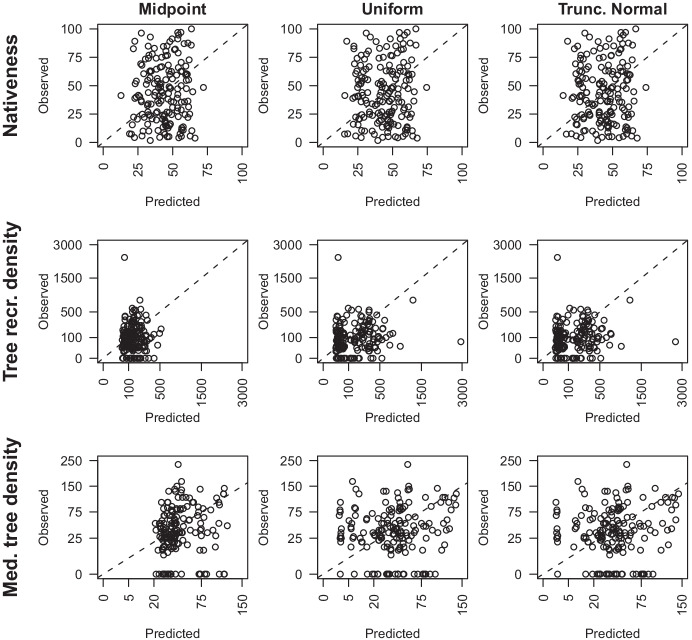
The predictions versus the observations of contemporary, objective response values for held-out data from 10-fold cross-validation (*n* = 170) of calibrated models of nativeness, tree recruitment density (stems per ha), and medium tree density (stems per ha). The three prior distributions, midpoint, uniform, and truncated normal, were assessed for each response variable. Tree density figures are plotted on a square root scale. Dashed lines represent perfect prediction, i.e., a 1:1 line

The cross-validation of the posterior of each response variable showed relatively poor predictive performance (Fig. 7). There was no relationship between predicted and observed values for any model alternative for nativeness and medium tree density models, and only a weak correlation for tree recruitment density dominated by outliers. The models tended to over-predict low values and under-predict high values, which is likely a result of the under-prediction of larger values within the calibration sub-model (Fig. 6).

#### Change sub-model

**Fig. 8:**
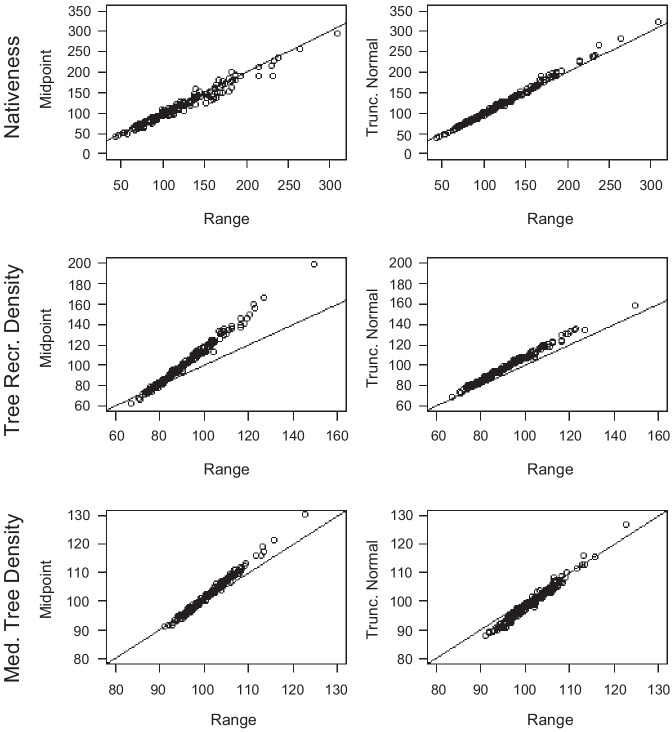
Model comparisons of percentage change, *α*, when midpoint, uniform, or truncated normal priors were used for calibration. Data shown for nativeness, tree recruitment density, and medium tree density (*n* = 174, 174, and 171, respectively). The solid line is a 1:1 line. Because the *α* values are transformed in the model and are difficult to interpret in that form, they were back-transformed and expressed as a percentage of the historical value, i.e., where 100% is no change

Modelled estimates of percentage change over time were highly correlated between the three prior forms used: midpoint, uniform, and truncated normal (Fig. 8). All three remaining prior forms produced highly correlated values for nativeness, whereas the Midpoint and Truncated Normal priors produced a slightly larger range of percentage change values across sites than Uniform priors for the tree density attributes.

#### Estimation of change

**Fig. 9:**
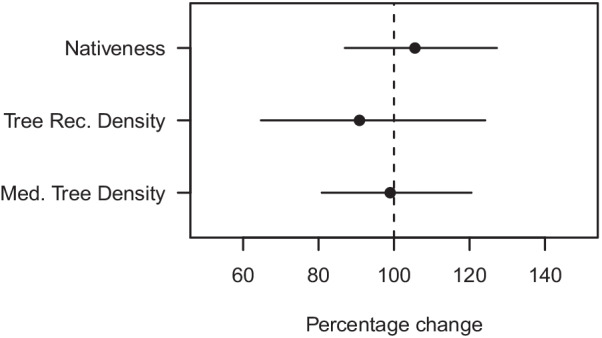
Grand mean, *μ*, percentage of historical value across all sites for each vegetation attribute from the calibration approach employing double sampling and the truncated normal prior. Error bars are 95% credible intervals. The dashed line is located at the 100% percentage change level, i.e., indicating no change

Based on the evaluation of the calibration sub-model and the overall model prediction of change, the use of a Truncated Normal prior appeared to perform as well or better than the other models in terms of making realistic predictions that matched the observations. The predictions of change were examined more closely in the Calibration models with the Truncated Normal prior in the calibration sub-model. Individual sites had predicted point estimates of percentage change ranging from 39.6 to 324.6, 69.1 to 158.6, and 88.2 to 126.7, for nativeness, tree recruitment density, and medium tree density respectively. The greater range of values for nativeness and tree recruitment reflects the capacity of those attributes to change in the 15-year interval between surveys. Sites were expected to vary in change due to the varied site history and local environmental attributes. Yet the uncertainty about any site’s change was great and the credible intervals for almost all the estimated changes overlapped 100 percent (no change). The mean change from the historical mean for each variable was always within ± 10% on average across all sites: nativeness (104.3% of historical mean), medium tree density (96.6%), and tree recruitment density (92.1%) (Fig. 9).

### Comparison between the two approaches

**Fig. 10:**
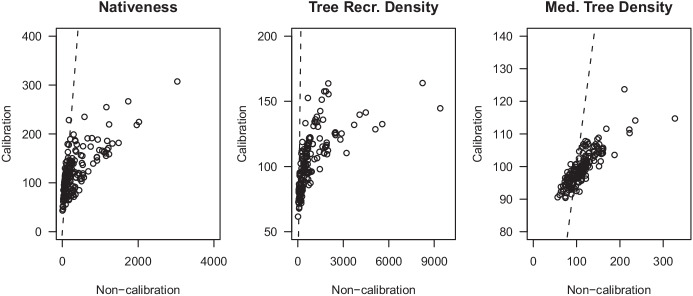
Comparisons between estimates of percentage change in each vegetation attribute from historical data (1994/1995) and contemporary data (2009/2010) using two alternative vegetation change estimation model forms: calibration and non-calibration. Both model forms used a Truncated Normal prior for historical values. Data are presented on a percentage scale where 100% is no change over time. The dashed line is a 1:1 line along which the estimates of change are equal between the two methods

Despite relatively weak predictive models, the calibration approach substantially outperformed the non-calibration approach for tree recruitment density but was only marginally better for medium tree density. Comparison of the models with truncated normal priors indicated that calibration models produced more realistic estimates of change over time than non-calibration alternatives, although both model forms produced relatively uncertain estimates. Non-calibration models for nativeness and tree recruitment density predicted some unrealistic overestimates of change (Fig. 10), which were predominantly those sites with historical scores of zero. Thus, for these sites, prediction using the Calibration model is clearly more accurate. For other sites, the non-calibration estimates were more comparable to those from the calibration. Predictions for medium tree density were more conservative; however, the non-calibration approach still predicted a much wider range of values (Fig. 10).

### Impacts of grazing using calibration approach using truncated normal priors

**Fig. 11:**
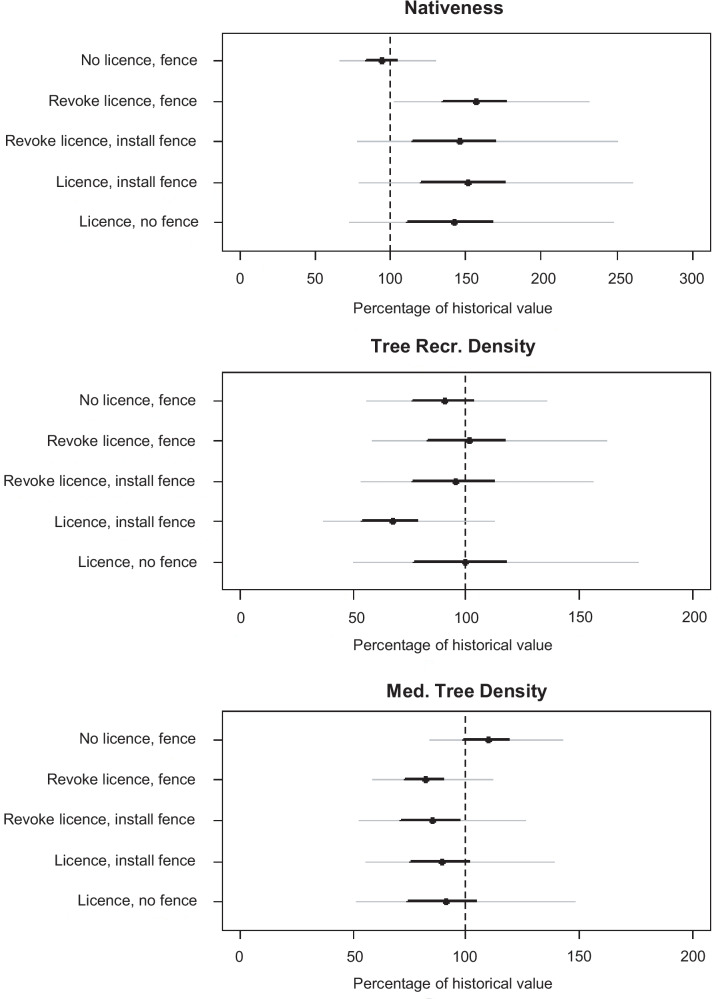
The predicted percentage of the historic modeled value for vegetation attributes under different grazing management classes, where 100% represents no change. Management classes are combinations of management status for each indicator (licenced or fenced) at the different time periods (1994/1995 and 2009/2010), for example “Revoke licence, fence” is the effect of sites that had licences revoked but were fenced in both periods. Results are for the Calibration approach using Truncated Normal priors. Points are the mean of change within each subset of management class. Thin error bars are 95% credible intervals; thick error bars are 50% credible intervals

**Fig. 12:**
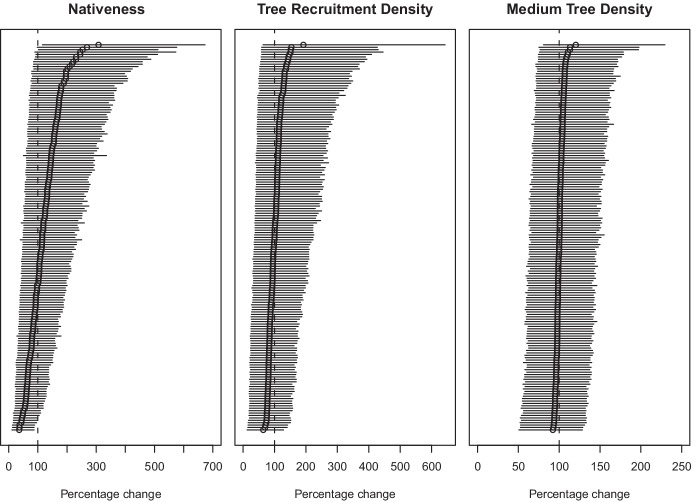
Ranked plot of the percentage change of the historical score in (**a**) nativeness, (**b**), tree recruitment density, and (**c**) medium tree density for each section. The dashed line indicates the zero change location (i.e., 100%). The circles are the mean estimates for change at each site with horizontal 95% credible intervals

Livestock grazing management influenced riparian vegetation attributes in the 15-year study period. Removal of a licence or installing a fence had positive and negative effects for different vegetation attributes (Fig. 11). Overall, the models predicted an increase in nativeness and a decrease in medium tree density across all sites. There was little overall trend in tree recruitment. However, the direction and magnitude of change in each vegetation attribute was highly variable between sites (Fig. 12), resulting in large uncertainty around the mean change across all sites (Fig. 9) and sites within management transition types (Fig. 11).

Vegetation nativeness can change due to changes to native, or exotic, vegetation cover, or both. Nativeness increased across most management transitions, although the sites with no change in management (i.e., “No licence, fence” and “Licence, no fence”) generally changed the least (Fig. 11). The smallest changes were predicted for fenced sites with no license. These sites were expected to be in the best condition due to the absence of grazing. The lack of change may be related to the starting condition of these sites because they generally had the highest historical scores for mostly native understorey cover. In contrast, the unfenced sites with a licence had the worst historical scores for mostly native understorey cover, which may be why they were predicted to increase despite no change in management.

There was little change on average in tree recruit density at fenced sites with no licence (Fig. 11). Sites with licence and had a fence installed were unexpectedly predicted to have a reduction in recruit density. Changes in tree recruits are a challenge to interpret due to there being two very different processes for a reduction in density; either the individuals have died, or they have grown beyond the size category. Medium tree density was not expected to change much in the study period. The overall reduction in predicted densities may be an artefact of the model or may reflect actual tree losses.

## Discussion

The approach of calibration via double sampling used in this study generally produced more realistic estimates for the reinterpreted historical data than the non-calibration approach. Without knowledge of the true values, we assume that these far more realistic estimates are more accurate and less biased. Use of these estimates has therefore likely resulted in more accurate estimates of change on average across the set of sites. Estimates of change in different vegetation attributes were highly uncertain due to the large differences in survey methods used in historical and contemporary surveys. This high uncertainty was expected and acknowledges the uncertainties that occur within and between datasets. The impact of livestock grazing was uncertain across all sites but the inferences of change as a result of removing a license or installing a fence were realistic and broadly matched our expectations.

### Non-calibration Bayesian reinterpretation

The non-calibration model approach of Bayesian reinterpretation is superior to the common alternative approach of choosing a midpoint. However, the non-calibration model approach of categorical data reinterpretation of Duncan and Vesk (2013) produced relatively poorly fitting models for the case study data and tended to overpredict values. Models with Truncated Normal priors marginally made the best predictions. The use of Normal priors was not suited to this study, due to the lack of an external data source to estimate the data distribution.

### Calibration via double sampling

The calibration via double sampling approach contained three sub-models. The calibration models performed relatively well, particularly when the truncated normal prior form from the Duncan and Vesk (2013) study was used. The midpoint prior produced less realistic estimates of change and in some cases a low correlation coefficient for change prediction. The relatively poor performance of the Midpoint has large implications for workers who use midpoint to reinterpret categorical data such as Braun-Blanquet cover scores (Gabrey & Afton, 2000; Gabrey et al., 2002; Naugle et al., 2000; Pahl et al., 1997; Poulin et al., 2002). The conversion of an estimated broad range (i.e., categorical estimate) to a point estimate (i.e., category midpoint) incorrectly implies a vastly increased precision of that estimate, which leads to unrealistic certainty of change.

Calibration of a historical dataset via double sampling is expensive due to the double sampling and more complex analysis, but there are many potential benefits. The Calibration approach described here can be used on any ordinal variables, regardless of how the data were collected, or how biased they are, or how coarsely surveyed, because this will be accounted for within the model and the uncertainty quantified. It is also possible to use it when there is no equivalent historical variable to match the contemporary variable, so long as there are other historical variables that are correlated with the contemporary variable. However, the more different the historical or subjective methods are from the contemporary methods, the more uncertain the predictions will be, and the more data required for effective analysis. The number of surveys and sites required for data calibration to be effective depends heavily on the heterogeneity of the vegetation between sites, the amount of change occurring, the resolution required to detect change, the local environmental conditions, and the vegetation type.

The large amount of uncertainty generated by the models should not be seen as a negative for a study of this type. A thorough accounting of the uncertainties within the datasets was precisely the objective of this study and encourages active discussion of their sources. The calibration via double sampling approach used here assumes that the survey biases from the calibration data are equivalent to those of the historical data. This assumption appears reasonable in this study because surveys were done by the same surveyor in each period; although biases for individual surveyors are unlikely to remain constant through time. Having the same surveyor for resurveys using an historical method is beneficial for the calibration model approach due to the high variation in visual estimation by different surveyors, e.g., Gorrod and Keith (2009). The use of different surveyors would therefore require incorporating observer error explicitly, in addition to the difference in methods, which will make the uncertainty greater.

The high degree of uncertainty and variation in predictions across sites results from uncertainties within the data that are propagated through the models, as well as the complexity of the study sites. There was over 200 km of stream length between the two most upstream and downstream sites. This large spatial scale encloses a variety of environmental and anthropogenic factors that are likely to influence the riparian vegetation, such as historical land use within and adjacent to frontages, soil, geomorphology, and hydrology. Incorporating each of these factors was outside of the scope of this study but we hope to explore these drivers further in the future.

### Impacts of livestock grazing in riparian areas

Grazing management actions of revoking a licence or installing a fence broadly matched our expectations within the study area. Predictions of a general increase in vegetation nativeness across sites should be encouraging for managers due to the high susceptibility of exotic species invasions in riparian areas due to high resource availability (Stohlgren et al., 1998; Swincer, 1986; Williams et al., 2008). However, the reduction in medium tree density is concerning and may require further investigation. Most trees within the study area are River Red Gums (*Eucalyptus camaldulensis*), which prefer periodic inundation (Horner et al., 2010; Wen et al., 2009). The contemporary survey occurred at the end of the “millennium drought” in Victoria, which severely reduced the tree health across much of the riparian landscape in south-eastern Australia (Doody et al., 2014). The slight increase in medium tree density at sites without a license in either survey may suggest that these ungrazed sites were more resilient to the drought conditions.

Tree seedlings were primarily River Red Gums and Grey Box (*E. microcarpa*). The former tend to recruit en masse following rain or flooding events and dense cohorts of recruits were observed in some sites, whereas the latter had more continuous but sporadic germination. The historical survey was performed following high rainfall in 1993, whereas the contemporary survey occurred at the end of the drought period (Bureau of Meteorology 2020), which may explain predicted lower density of recruits in some sites within the contemporary study area.

### Guidance for application

Employing either the calibration or non-calibration approaches used here to account for the differences in datasets can account for some of the variation that occurs within and between different datasets. Doing this can enable comparisons between disparate datasets over long timeframes or large spatial scales. Without some approach to explicitly account for differences in data collection methods, such as those explored in this study, the comparisons of data are likely to be less accurate and less precise. With any approach, the more different the datasets are, the more uncertainty there will be. While it is crucial for researchers to adopt approaches to account for differences in data, this challenge highlights the importance of consistency in data collection methods.

A key advantage of the calibration approach described here is that it can be used for any type of data, e.g., categorical, count, cover, quantitative or qualitative. However, the performance of the approach will largely depend on the strength of correlations between data used in the calibration. The calibration approach is also the most expensive option because of the additional field data collection costs associated with double sampling. With fixed or limited budgets, this would require a trade-off between sampling more sites once, versus fewer sites with a double sampling subset. The result of the trade-off will be different for different studies, budgets, objectives, and sampling approaches but is likely to be dictated by a need for data accuracy (calibration) versus data volume (non-calibration).

Based on the outcomes of this study and other relevant studies, below is a simple and clear summary below of the important outcomes and some guidance on application. Firstly, the following recommendations may be considered when attempting to reinterpret coarse historical data:

1. Do not use category midpoints. This implies a level of precision never specified by the original observations, and it is biased with respect to the underlying data distributions (see also McNellie et al., 2019).
2. Of the prior forms examined, use the truncated normal. In general, it outperformed alternatives, though we urge further work on admitting possible misclassification error among the subjective classes.
3. Consider double sampling and calibration. The benefits to precision will depend monotonically on (a) the strength of the calibration relationship and on (b) the coverage of the broader dataset (sample size and stratification across the response and predictors of the dataset).

Secondly, given the increased effort and resources to conduct calibration by double sampling, this approach will not always be feasible or desirable. There are therefore a set of situations or circumstances where this approach may be most valuable:

1. Where there is a need for high precision. A need for high precision may result from an expected or known small magnitude of response, or alternatively if a very low tolerance of uncertainty is required. In either case, this approach is an effective way of improving precision above many alternative options.
2. Where sufficient resources are available. Due to the additional survey and analysis effort, the costs of calibration by double sampling are greater than many alternatives. This is a problem where resources are limited. Importantly, the amount of resources required will depend on the situation; if a large calibration dataset is required because of inherent variability in the data, or because there is a week calibration relationship between datasets, then the costs will be greater.
3. Where survey methods can be reliably applied. Historical data methods may be difficult to reapply if the method was not clearly described or there are no available practitioners with method experience. In this case, there may be errors or biases in the application of the calibration surveys that are not the same as those in the historical data, which may result in poor calibration relationships with historical data regardless of relationships with new data.

## Data Availability

Data and code relating to this manuscript will be made available via a public Open Science Framework repository upon acceptance.

## Appendix

### Model structure description and interim outputs

The calibration model is used to predict the expected probability of success for historical data by estimating the number of hits from the same number of points using historical covariates and other associated predictor variables.

#### Bayesian informative priors

Four different Bayesian informative prior options were compared within the study. Each prior is applied to the categorical data predictor variables, X_*k*_. For the “midpoint” method, the categorical predictors were set to the midpoint values of each category and were included in the model as data. Ranges of categorical variables (“uniform”) were delimited by the upper and lower limits of the category, L_*k*_,U_*k*_, and distributions (“normal”) were centered around a mean, *z*, and standard deviation, σ, estimated from the full contemporary dataset. Historical predictors, and therefore the range and mean values for their priors, were log-transformed densities, and cloglog-transformed cover proportions.

$$Uniform:\;\;\;\;\;\;\;\;\;\;\;\;\;X_k\sim Uniform(:L_k,U_k)$$

$$Normal:\;\;\;\;\;\;\;\;X_k\sim Normal(z,\sigma)$$

$$Truncated\;Normal:\;\;\;\;\;\;\;\;X_k\sim Normal(z,\sigma)\;\left|\;(L_k,U_k)\right.$$

$$Minpoint:\;\;\;\;\;\;\;\;\;X_k=M_k$$

For the subjective historical surveys, tree recruitment densities were recorded in categories covering 0, < 40, and > 40 recruits per hectare, whereas for medium-sized trees the categories were 0, < 50, and > 50 trees per hectare. We converted the categories to ranges with minimum and maximum values. For the uniform and truncated normal methods, we selected minimum values slightly above zero as zero is undefined for a log-transformation. The maximum values of the largest categories were derived from the largest observed values seen in the contemporary survey data. For the midpoint method, we used the midpoint of the middle class, but for the upper class, which is open ended, we took the midpoint of its lower bound and the maximum derived from the contemporary surveys (150 trees per hectare for medium trees, and 500 recruits per hectare for tree recruitment). The midpoint of the lowest class was set at 2 for both types of tree densities, which lies outside the ranges specified for the Uniform method, as this value worked well as a substitute for the lowest category of zero.

#### Calibration sub-model

The contemporary response variable “nativeness” was derived from point quadrat sampling along transects, is in the form of number of “hits,” or success (where a life form is present at a point) from the total number of “points,” or trials. The calibration sub-model for nativeness took the form:

$$R_k\sim Binomial(q_k,m_k)$$

$$cloglog(q_k)=a+bX_{1k}+cX_{2k}+\eta_k,\;\;\;\;\;\;\;\;For\;k,\;1...60$$

where for the *k*th site the objective method data, *R*_*k*_, was binomially distributed with probability, *q*_*k*_*,* and sample size, *m*_*k*_. The cloglog transformed probability of *q*_*k*_ is a linear function of predictor variables, X_1*k*_, X_2*k*_, with coefficients, *b*, *c*, using subjective methods from calibration data, an error term describing discrepancy of the sites from the prediction, *η*_*k*_, and an intercept, *a*.

#### Prediction sub-model

The prediction sub-model used the same function, intercept and coefficients but using historical and site predictor data, X_1β*i*_, X_2β*i*_, with coefficients, *b*, *c*, to predict the cloglog transformed probability of a hit, *r*_*i*_ (Fig. 1).

The site error term, *ε*_*i*_, was modelled as normally distributed with the mean, 0, with the same precision, *τ*_*1*_*,* as derived from the calibration model.

$$c\mathrm{loglog}(r_i)=a+bX_{1\beta i}+cX_{2\beta i}+\varepsilon_i,\;\;\;\;\;\;\;For\;i,\;1...180\;(i.e.,\;the\;historical\;data\;set,\;180\;sections)$$

$$\varepsilon_i\sim Normal\;(0,\tau_1)\;\;\;\;\;\;\;\;\;\;Where\;\tau_1=1/\sigma_1^2\;and\;\sigma_1\sim Uniform(0,100)$$

#### Change sub-model

For a given response variable, the current probability of a hit, *p*_*i*_, is equal to the calibrated past probability, *r*_*i*_, plus some degree of change, *δ*_*i*_ (Fig. 1). Because seasonal variation influences many vegetation attributes, the difference in time of year between surveys, *T*_*i*_, was included in the model for change over time with coefficient, *d*. The raw change, α_*i*_, is that remaining after the seasonal influence is removed, and is normally distributed with the mean change, *μ*, and the precision of change, *τ*_*2*_ estimated from the data. Seasonality, *T*_*i*_, was excluded from models of variables that are unlikely to change in due to seasonal variation, e.g., tree density.

$$c\mathrm{loglog}(p_i)=c\mathrm{loglog}(r_i)+\delta_i\;\;\;\;\;\;\;\;For\;i,\;1...180$$

$$\delta_i=a_1+{dT}_i$$

$$a_i\sim Normal\;(\mu,\tau_2)\;\;\;\;\;\;\;\;\;\;Where\;\tau_2=1/\sigma_2^2\;and\;\sigma_2\sim Uniform(0,100)$$

#### Non-calibration (Bayesian reinterpretation) approach

This approach was based on the method of Duncan and Vesk (2013). Unlike the Calibration approach, the response variable for nativeness at the *i*th site, *Y*_*i*_, was a normally distributed cloglog transformed proportion of cover with mean, *v*_*i*_*,* and standard deviation, *w*_*i*_. The standard deviation was set at 100 to indicate an uncertain mean. Proportions of cover were derived from the number of vegetation hits from the number of points sampled at each site. The response data for medium tree density and tree recruitment density were again modeled as a Poisson distributed variable, where *Y*_*i*_, are the number of trees counted at the *i*^th^ site. The mean count, λ_*i*_, is a function of the density, *m*_*i*_, and the site area, *s*_*i*_.

$$Y_i\sim Normal(v_i,w_i)\;\;\;\;\;\;\;\;For\;i,\;1...180$$

$$Y_i\sim Poison(\lambda_i)$$

$$\log(\lambda_i)=\log(m_i)+\log(s_i)$$

We used a cloglog link function to transform the proportions, with normally distributed errors. Counts were Poisson distributed and log transformed densities were modelled using a log link function to estimate change. The contemporary response variable, *Y*_*i*_, is then a function of the past value, *r*_*i*_, plus some change, δ_*i*_.

$$Nativeness:\;\;\;\;\;\;cloglog(Y_i)=c\mathrm{loglog}(r_i)+\delta_i\;\;\;\;\;\;\;\;For\;i,\;1...180$$

$$Tree\;density:\;\;\;\;\;\log(Y_i)=log(r_i)+\delta_i$$

Because projective foliage cover of understorey life forms can vary seasonally, the change in cover was modelled as a function of true change, *α*_*i*_ and the difference (in days) in the time of year between the subjective and objective surveys, D_*i*_, with coefficient, β.

$$\delta_i=a_i+\beta^\ast D_i$$

$$a_i\sim Normal\;(\mu,\tau_2)\;\;\;\;\;\;\;\;\;\;Where\;\tau_2=1/\sigma_2^2\;and\;\sigma_2\sim Uniform(0,100)$$

## Code availability

The research was conducted using software programs and custom code as specified within the manuscript methods.
